# Associations between income and coping strategies among households at risk of food insecurity in a high cost-of-living region

**DOI:** 10.1093/haschl/qxag076

**Published:** 2026-03-27

**Authors:** Ana Altares, Laura L Bellows, Rebecca Cleary, Carrie Chennault, Megan P Mueller

**Affiliations:** College of Health Solutions, Arizona State University, Phoenix, AZ 85004, United States; Division of Nutritional Sciences, Cornell University, Ithaca, NY 14853, United States; Department of Agricultural Resource and Economics, Colorado State University, Fort Collins, CO 80523, United States; Department of Anthropology and Geography, Colorado State University, Fort Collins, CO 80523, United States; Department of Food Science and Human Nutrition, Colorado State University, Fort Collins, CO 80523, United States

**Keywords:** food security, food assistance, coping strategies, community health, social determinants of health

## Introduction

Food insecurity, the limited access to affordable, safe, and nutritious food, disproportionately affects individuals living in poverty and is associated with poorer health outcomes and higher health care spending.^1^ While the Supplemental Nutrition Assistance Program (SNAP) helps reduce food insecurity; rising cost-of-living and geographic variation in affordability mean that benefits often fall short of household need.^2^ In 2023, nearly half of food-insecure households earned above 130% of the federal poverty level (FPL), the national SNAP eligibility cutoff. These households may turn to coping strategies such as (1) accessing charitable food, including food pantries, and (2) economic tradeoffs, such as choosing between food and medicine or utilities.^1^

Most research has focused on families earning below 130% FPL, leaving limited understanding of how households with income above 130% FPL navigate resource constraints. We examined differences in charitable food reliance and tradeoffs between households at risk of food insecurity with incomes of 130% or less of the FPL and 131%–200% FPL living in Colorado's rural, high cost-of-living Parachute to Aspen Corridor (PAC). Marked by extreme housing costs and economic disparities, this region provides a relevant setting to examine food insecurity among households with income above federal SNAP thresholds.

## Methods

We conducted a cross-sectional survey of PAC residents from December 2022 to March 2024. Participants were 18 years or older, lived or worked in the PAC region, and screened at risk of food insecurity using the Hunger Vital Sign screener (Children’s Health Watch). Surveys were conducted online, by phone, or paper in English or Spanish.

The outcomes of interest were well-documented food-insecurity coping mechanisms: reliance on charitable food and tradeoffs. Reliance on charitable food was determined via how much total food came from food pantries, soup kitchens, free foods from family/friends, or “other” charitable food sources in the past 30 days. Response categories were “none,” “a small portion,” “about half,” or “the majority.” The frequency of economic tradeoffs was measured separately between food and each of these basic needs (medicine, housing, utilities, childcare, transportation, and education) within the last 30 days. Response categories were “never,” “occasionally,” and “frequently.”

The primary predictor of interest was categorized using the 2022 poverty guidelines: 130% FPL or lower and 131%–200% FPL. Covariates included overall scores for barriers to and stigma and shame around charitable food (charitable food models only); household resilience, financial literacy (tradeoffs models only); use of federal assistance; and sociodemographic characteristics (age, sex, race/ethnicity, education, household size, marital status, employment, housing status, and degree of food security). Additional details on how variables were measured can be found in Table S1 and the study by Altares (2025).^3^

Generalized ordinal logistic regression (GOLR) models evaluated the odds of obtaining different amounts of food from each charitable food source and frequency of engaging in each tradeoff among participants with incomes of 130% FPL or lower and 131%–200% FPL. The GOLR produces multiple binary comparisons of lower vs higher levels of coping strategy variables.

Since SNAP eligibility guidelines are complex and dependent on household circumstances (ie, deductions and broad-based categorical eligibility set at 200% FPL), a sensitivity analysis examined income as an ordinal variable (instead of dichotomous), for both charitable food and tradeoff models.

## Results

The final analytic sample included 1021 participants. Descriptive differences by income group can be found in Table S1. Compared with participants at 130% FPL or lower, participants with incomes of 131%–200% FPL had approximately 2 times the odds of obtaining approximately half or more of their total food from food pantries (odds ratio [OR] = 1.84–2.43) and soup kitchens (OR = 1.74–1.93; *P* < .05 for all tests) (Table 1).

**Table 1.** Differences in the odds of utilizing coping strategies such as obtaining household food from charitable food assistance or engaging in economic tradeoffs between food and other basic needs for participants at ≤130% FPL and those at 131%–200% FPL.

	Portion of total household food obtained from charitable food		
	**≥ A small portion** **vs none**	**≥ About half vs** **≤ a small portion**	**The majority** **vs ≤ about half**
Charitable food assistance source			
Food pantries^a^	0.99 (0.69, 1.43)	1.84 (1.39, 2.43)***	2.43 (1.71, 3.44)***
Soup kitchens^a^	0.75 (0.56, 1.00)	1.74 (1.21, 2.48)**	1.93 (1.12, 3.32)*
Free food (family/friends)	1.18 (0.91, 1.54)	1.18 (0.91, 1.54)	1.18 (0.91, 1.54)
Other CFA	1.35 (0.99, 1.84)	1.35 (0.99, 1.84)	1.35 (0.99, 1.84)

	Frequency engaging in tradeoffs between food and basic needs	
	**≥ Occasionally (vs never)**	**Frequently (vs ≤ occasionally)**
Economic tradeoffs		
Medicine	1.56 (1.17, 2.08)**	1.56 (1.17, 2.08)**
Housing	0.96 (0.85, 1.08)	0.96 (0.85, 1.08)
Utilities^a^	0.86 (0.75, 0.99)*	1.29 (1.12, 1.50)**
Childcare	1.18 (1.04, 1.33)**	1.18 (1.04, 1.33)**
Education^a^	1.25 (1.10, 1.42)**	1.47 (1.25, 1.73)***
Transportation^b^	1.23 (1.07, 1.41)**	1.23 (1.07, 1.41)**

Abbreviations: CFA, charitable food assistance; GOLR, generalized ordinal logistic regression; OR, odds ratio.

Values are GOLR ORs (95% CI). Odds ratios represent cumulative GOLR thresholds. Models were adjusted for age, sex, education, employment, housing status, marital status, race/ethnicity, and current receipt of federal assistance. Charitable food models were also adjusted for charitable food barrier score and stigma-shame score. Economic tradeoff models were also adjusted for household resilience and financial literacy.^3^ * *P* < .05; ** *P* < .01; *** *P* < .001.

^a^The assumption for proportional odds for the primary variable, income above/below 130% FPL, were violated, yielding variable ORs for each level of the outcome variables food pantries, soup kitchens, utilities, and education.

^b^Sample size for tradeoffs between food and transportation was limited to *n* = 764 due to missing data.

Participants with incomes 131%–200% FPL were also significantly more likely to engage in tradeoffs between food and medicine (OR = 1.56), childcare (OR = 1.18), education (OR = 1.25, 1.47), and transportation (OR = 1.23; *P* < .05 for all tests) “occasionally” or more as well as “frequently” or more (Table 1). For utilities, they were more likely to report tradeoffs between food and utilities “frequently” or more (OR = 1.29), but less likely to report tradeoffs “occasionally” or more (OR = 0.86; *P* < .05 for all tests).

Sensitivity analyses using income as an ordinal variable were consistent. As income increased, (1) reliance on charitable food increased (Table S2) and (2) tradeoffs between food and other basic needs increased (Table S3).

## Discussion

These findings indicate that, in a high cost-of-living rural region, food-insecure households with more income were more likely than households at 130% FPL or lower to rely on charitable food and to engage in tradeoffs between food and other basic needs. These results challenge assumptions that households with relatively higher incomes are less vulnerable to food insecurity and highlights limitations of existing support systems, particularly in high cost-of-living settings.^4^ Findings also highlight the interconnected nature of food and other basic needs insecurity.

Greater reliance on coping strategies among households at 131%–200% FPL may reflect program eligibility cliffs and the inadequacy of benefit levels relative to local costs. Significant tradeoffs between food and transportation, childcare, education, medicine, and utilities suggest that financial strain extends beyond food to expenses that are fundamental to economic stability. Tradeoffs with housing did not differ significantly by income group, suggesting that housing pressures may be widespread across households at 200% FPL or lower. While SNAP provides critical food assistance, its benefits are restricted to food purchases and may not address competing financial demands. Supports such as the Child Tax Credit may offer more flexible help to meet the multidimensional hardships reflected in these findings.

Limitations included recruitment through community partners that may have overrepresented charitable food recipients, although social media outreach likely broadened participation. SNAP eligibility was also approximated using only income thresholds.

Strengths include a large and diverse sample that reflects national food insecurity rates among SNAP-eligible households and regional demographics for sex, education, and Latino/Hispanic ethnicity.^5^ A community-engaged, multilingual, and multimodal survey approach enhanced accessibility.

Policy approaches that expand cross-sector supports, such as the Child Tax Credit, and adjust SNAP eligibility thresholds or benefit levels to account for regional cost-of-living differences may better align assistance with household need. Future research should examine how coping strategies vary across contexts and whether policy reforms can reduce reliance on charitable food assistance programs.

## Supplementary Material

qxag076_Supplementary_Data

## Data Availability

The data that support the findings of this study are not publicly available due to privacy restrictions but may be available from the corresponding author upon reasonable request and with appropriate approvals.

## References

[1] Seligman HK, Berkowitz SA. Aligning programs and policies to support food security and public health goals in the United States. Annu Rev Public Health. 2019;40(1):319–337. 10.1146/annurev-publhealth-040218-044132 30444684 PMC6784838

[2] Massachusetts Institute of Technology. Data from: Living wage data sourced from the Living Wage Institute . 2025. https://livingwage.mit.edu/.

[3] Altares A . Coping Strategies Among Food Insecure Households Above and Below SNAP Eligibility Guidelines in a High Cost-of-Living Region. Colorado State University; 2025.

[4] Gundersen C, Ziliak JP. Food insecurity research in the United States: where we have been and where we need to go. Appl Econ Perspect Policy. 2018;40(1):119–135. 10.1093/aepp/ppx058

[5] US Census Bureau and Bureau of Labor Statistics . Data from: Current Population Survey (CPS) Quick Facts. Accessed December 2024. https://www.census.gov/quickfacts/fact/table/garfieldcountycolorado,pitkincountycolorado,eaglecountycolorado/PST045225

