# Supplementary material for: Associations between income and coping strategies among households at risk of food insecurity in a high cost-of-living region
Source: Health Aff Sch. 2026 Mar 27;4(4):qxag076. doi: 10.1093/haschl/qxag076 (PMC13070723; doi:10.1093/haschl/qxag076)
Supplement: qxag076_Supplementary_Data [file qxag076_supplementary_data.zip › Supplemental Materials.docx]

## Supplemental Materials

**Supplemental Table 1.** Differences in sociodemographic characteristics and other health-related behaviors for participants, overall and by income.

|  | Total  (n=1,021, 100%),  [% or Mean (SD)] | <130% FPL  (n=610, 59.7%),  [% or mean (SD)] | | | 131-200% FPL (n=411, 40.3%),  [% or mean (SD)] | | |
| --- | --- | --- | --- | --- | --- | --- | --- |
| **Race/ethnicity** |  | | |  | | |  |
| White/Caucasian | 37.3 | | 37.9 | | | 36.5 | |
| Latino/Hispanic | 22.9 | | 22.0 | | | 24.3 | |
| Black/African American | 14.7 | | 18.4*** | | | 9.3*** | |
| Other^a^ | 25.1 | | 21.8** | | | 29.9** | |
| **Sex** | | | | | | | |
| Female | 57.0 | | 50.8*** | | | 66.2*** | |
| Male | 40.9 | | 47.1*** | | | 31.9*** | |
| Other | 2.0 | | 2.1 | | | 2.0 | |
| **Age (years)** | 39.2 (9.6) | | 39.5 (9.4) | | | 38.6 (9.9) | |
| **Education**^b^ | | | | | | | |
| No high school degree | 13.0 | | 15.6** | | | 9.3** | |
| High school degree | 46.3 | | 46.9 | | | 45.5 | |
| College/Graduate degree | 40.7 | | 37.5* | | | 45.3* | |
| **Household Size** | 3.2 (1.4) | | 3.1 (1.4) | | | 3.2 (1.4) | |
| **Married** | 51.8 | | 49.5 | | | 55.1 | |
| **Income Group (% FPL)** | | | | | | | |
| < 130% FPL | 59.7 | | - | | | - | |
| 131 – 185% FPL | 36.7 | | - | | | - | |
| 186 – 200% FPL | 3.5 | | - | | | - | |
| **Unemployed** | 15.0 | | 13.1* | | | 17.8* | |
| **Temporary Housing** | 27.3 | | 27.9 | | | 26.5 | |
| **Language Spoken** |  | |  | | |  | |
| English | 70.4 | | 74.3** | | | 64.7** | |
| Spanish | 18.7 | | 17.9 | | | 20.0 | |
| Bi- or Multilingual | 10.9 | | 7.9*** | | | 15.3** | |
| **Food Insecurity (total household)** | | | | | | | |
| Marginal Food Security | 7.9 | | 5.6** | | | 11.4** | |
| Low Food Security | 16.3 | | 14.3* | | | 19.2* | |
| Very Low Food Security | 75.8 | | 80.2*** | | | 69.3*** | |
| **Federal Assistance Recipients** | | | | | | | |
| SNAP | 60.5 | | 63.2* | | | 56.5* | |
| Any federal assistance ^c^ | 76.0 | | 78.8* | | | 72.0* | |
| **Charitable Food Assistance Barrier Score** | 4.3 (3.6) | | 4.6 (3.8) ** | | | 3.9 (3.3) ** | |
| **Stigma/shame Score** | 2.1 (2.4) | | 2.0 (2.3) | | | 2.1 (2.5) | |
| **Financial Literacy Score** | 6.0 (4.0) | | 6.1 (4.0) | | | 5.7 (4.1) | |

*Note:* SD – standard deviation. FPL = federal poverty level. AI/AN = American Indian/Alaska Native. Asian includes Asian, South Asian, and Pacific Islander identified participants. SNAP = Supplemental Nutrition Assistance Program. Categorical variables analyzed using chi–square test. Continuous variables analyzed using *t*-test. Food security was based on the USDA 10-18-item survey. Charitable Food Assistance Barrier Score was based on a scale 0 – 11. Stigma/shame Score was based on a scale 0 – 10. Financial Literacy Score was based on a scale 0 – 16.

a. “Other” races include Multiracial, American Indian/Alaska Native, Asian, and Other.

b. Education levels were condensed as follows: No high school degree (eighth grade or less & some high school), High school degree (high school diploma or GED & some college), College/graduate degree (college & graduate school or professional degree).

c. Any federal assistance included Supplemental Nutrition Assistance Program (SNAP), Special Supplemental Nutrition Program for Women, Infants, and Children (WIC), Supplemental Security Income (SSI), Temporary Assistance for Needy Families (TANF), meal services targeting older adults.

* *p*<.05; ** *p*<.01***; *p*<.001

## Supplemental Table 2. Differences in use of charitable food assistance by increasing income level.

|  | **GOLR Odds Ratios, Adjusted (95% Confidence Interval)** | | | | | |
| --- | --- | --- | --- | --- | --- | --- |
|  | | Portion of total household food obtained from charitable food | | | | |
|  | | > A Small portion  vs None | | > About half vs  < A small portion | | The majority  vs <About half |
| **Charitable Food Assistance Sources** | | |  | |  | |
| Food pantry^a^ | | 1.02 (.90, 1.16) | | 1.20 (1.08, 1.34) ** | | 1.53 (1.33, 1.75) *** |
| Soup kitchen^a^ | | 0.84 (.75, .94) ** | | 1.26 (1.1, 1.44) ** | | 1.30 (1.07, 1.58) ** |
| Free food (Family/friends) | | 1.10 (1.00, 1.22) | | 1.10 (1.00, 1.22) | | 1.10 (1.00, 1.22) |
| Other CFAs^a^ | | 1.14 (1.01, 1.29) * | | 1.16 (0.98, 1.38) | | 1.79 (1.37, 2.34) *** |

*Note:* GOLR = Generalized Ordinal Logistic Regression. In this sensitivity analysis, income was used as an ordinal variable to show similar trends in the main model using income as a binary indicator variable. Models were adjusted for age, sex, education, employment, housing status, marital status, race/ethnicity, current receipt of federal assistance, charitable food assistance barrier score, and stigma-shame score. ^3^

a. The assumption for proportional odds for the primary variable, income, were violated, yielding variable odds ratios for each level of the outcome variables food pantries and soup kitchens.

* *p*<.05; ** *p*<.01; *** *p*<.001

##

## Supplemental Table 3. Differences in frequency of economic tradeoffs by increasing income level.

|  | **GOLR Odds Ratios, Adjusted (95% Confidence Interval)** | | | |
| --- | --- | --- | --- | --- |
|  | | Frequency of engagement in tradeoffs between food and basic needs | | |
|  | | > Occasionally  (vs Never) | | Frequently  (vs < Occasionally) |
| **Economic Tradeoffs** | | |  | |
| Medicine | | 1.17 (1.05, 1.31) ** | | 1.17 (1.05, 1.31) ** |
| Housing | | 1.01 (.91, 1.13) | | 1.01 (.91, 1.13) |
| Utilities ^a^ | | 0.96 (0.85, 1.09) | | 1.27 (1.12, 1.45) *** |
| Childcare | | 1.18 (1.06, 1.32) ** | | 1.18 (1.06, 1.32) ** |
| Education ^a^ | | 1.19 (1.06, 1.34) ** | | 1.44 (1.25, 1.66) *** |
| Transportation | | 1.20 (1.06, 1.36) ** | | 1.20 (1.06, 1.36) ** |

*Notes:* GOLR = generalized ordinal logistic regression. In this sensitivity analysis, income was used as an ordinal variable to show similar trends in the main model using income as a binary indicator variable. Models adjusted for age, sex, education, employment status, housing status, marital status, race/ethnicity, current use of federal assistance, household resilience, and financial literacy. ^3^

a. The assumption for proportional odds were violated, yielding variable odds ratios for each level of the outcome variables food pantries and soup kitchens.

b. Sample size for tradeoffs between food and transportation was limited to n=764 due to missing data.

* *p*<.05; ** *p*<.01, ****p*<.001
